# *In vivo* detection of nanometer-scale structural changes of the human tympanic membrane in otitis media

**DOI:** 10.1038/s41598-018-26514-1

**Published:** 2018-06-08

**Authors:** Roshan Dsouza, Jungeun Won, Guillermo L. Monroy, Malcolm C. Hill, Ryan G. Porter, Michael A. Novak, Stephen A. Boppart

**Affiliations:** 1 Beckman Institute for Advanced Science and Technology, University of Illinois at Urbana-Champaign, Urbana, Illinois USA; 2 Department of Bioengineering, University of Illinois at Urbana-Champaign, Urbana, Illinois USA; 3 Department of Pediatrics, Carle Foundation Hospital, Urbana, Illinois USA; 4 Carle-Illinois College of Medicine, University of Illinois at Urbana-Champaign, Urbana, Illinois USA; 5 Department of Otolaryngology, Carle Foundation Hospital, Urbana, Illinois USA; 6 Department of Electrical and Computer Engineering, University of Illinois at Urbana-Champaign, Urbana, Illinois USA

## Abstract

Otitis media (OM) is a common ear infection and a leading cause of conductive hearing loss in the pediatric population. Current technologies such as otoscopy, pneumatic otoscopy, tympanometry, and acoustic reflectometry are used to diagnose OM, which can reasonably diagnose the infection with a sensitivity and specificity of 50–90% and 60–90%, respectively. However, these techniques provide limited information about the physical architecture of the tympanic membrane (TM), or what may lie behind it. Here, we report the detection of nanometer-scale structural changes of the TM using nano-sensitive optical coherence tomography (nsOCT). In total, an image dataset from 65 pediatric subjects from three different groups (normal, acute OM, and chronic OM) and with longitudinal image-based analysis of ear infections were included in this study. The nsOCT data were correlated with physician diagnosis and with OCT thickness measurements and were found to be in good agreement with these results. We report that nsOCT detects *in vivo* structural deformations of the TM earlier than OCT alone, and enhances the detection sensitivity of OCT measurements. This unique technique for early detection of nano-scale structural modifications in the TM has the potential to aid in our understanding of microbiological effects, and possibly for early diagnosis and more effective treatment of OM.

## Introduction

The tympanic membrane (TM) is a thin, circular layer of tissue that separates the outer and middle ear. It consists of three main layers: an outer epithelium, a middle fibrous lamina propria, and an inner mucosal layer. Radial and circular fibers give the membrane stiffness and tension. Submicroscopic details of the human TM using transmission electron microscopy (TEM) and scanning electron microscopy (SEM) reveal that the layers of the TM are periodic structures^1^. These periodic inner structures become deformed or aperiodic in various clinical conditions, such as in otitis media (OM)^2^ and the associated inflammation. OM is a middle-ear infection that involves complex biological processes and changes, including the later development of middle-ear biofilms that serve as reservoirs for antibiotic-resistant bacteria^3^. Nearly eighty percent of children in the US will develop an ear infection by the age of three^4^, making this one of the most highly prevalent childhood diseases. OM is often accompanied by fluid accumulation (an effusion) in the middle ear cavity, which is diagnosed as OM with effusion (OME). The infection is caused by one or several bacteria/viruses in the middle-ear, leading to erythema and inflammation of the ear canal and the TM, along with the associated “fullness” and pain. Diagnosis of such ear infections is generally carried out using an otoscope, where the physician examines the outer surface of the TM for signs of redness, opacity, the presence of fluid behind the TM, and with the use of pneumatic otoscopy, TM mobility. These subjective observations are subsequently used to dictate treatment, which often includes oral antibiotics. However, the sensitivity and specificity of traditional otoscopy for determining the presence of OM is reported to be only 74% and 60%, respectively, and is also biased on the experience of the physician^5,6^. Furthermore, the diagnostic accuracy between OME and acute otitis media (AOM) using a video otoscope among otolaryngologists, pediatricians, and general practitioners was 74%, 51%, and 46%, respectively^7^. This subjective observation from otoscopy can lead to incorrect diagnoses, over prescription of antibiotics for treatment, and incorrect prognoses. Other techniques such as pneumatic otoscopy, acoustic reflectometry, and tympanometry are used to diagnose OME, but provide limited depth-resolved information about the structural changes in the TM or the presence of any biofilm behind the TM.

Optical coherence tomography (OCT) is an established optical imaging modality for biomedical research and clinical applications that is based on low-coherence interferometry^8^. OCT performs high-resolution, depth-resolved *in vivo* imaging of biological structures by measuring the echo-time delay, magnitude, and phase of backscattered light. Since its first demonstration in 1991, OCT has had its most clinical success in ophthalmology, where it provides structural and quantitative information of the eye that is difficult to obtain by any other imaging modality. OCT has also found clinical applications in cardiology, dermatology, gastroenterology, and oncology^9^, to name a few. OCT has been used to investigate structural and dynamic changes of the ear, both *ex vivo* and *in vivo*^10,11^. With the development of a compact handheld scanner and a portable system, the practical use of OCT has been demonstrated in clinical settings^12,13^. These studies demonstrated that OCT could provide quantitative structural information of the TM, such as a thickness measurement, determining the presence and thickness of any middle-ear biofilm, determining the presence and optical and mechanical properties of any middle ear effusion, and detecting subtle micron-scale pneumatic-induced displacements of the TM^14–19^. Despite its high spatial and temporal resolution and high-sensitivity detection, the structural sensitivity of OCT has been limited to the micron scale. However, most biological processes, including the development of middle-ear infections and bacterial biofilms, begin at the nano-scale level^20^. The laminar structures of the TM undergo significant structural changes during disease progression, as evidenced by comparative histopathology of a normal TM with those under the conditions of AOM and OME^2^. Hence, detecting these nano-structural changes *in vivo* at early stages not only offers the potential to enhance the sensitivity of the OCT measurements, but also helps to elucidate the changes that occur in the infectious process and the establishment of a bacterial biofilm in the middle ear.

In this investigation, we have implemented nano-sensitive optical coherence tomography (nsOCT) to detect the structural changes in the human TM with nano-scale sensitivity and accuracy^21,22^. We report *in vivo* nano-scale structural changes in the TMs of a cohort of pediatric human subjects, as well as track the structural changes during a longitudinal study of subjects undergoing surgical intervention (tympanostomy tube placement in the TM) for chronic OM. Our results demonstrate that nsOCT detects structural changes at an earlier stage, compared to standard OCT, and that the application of this method could provide new insight into the microbiological and inflammatory effects on human tissue, as well as potentially increase the sensitivity of OCT for diagnostic assessments and clinical decision making in this highly prevalent disease.

## Results

### Displacement stability measurements

**Figure 1:**
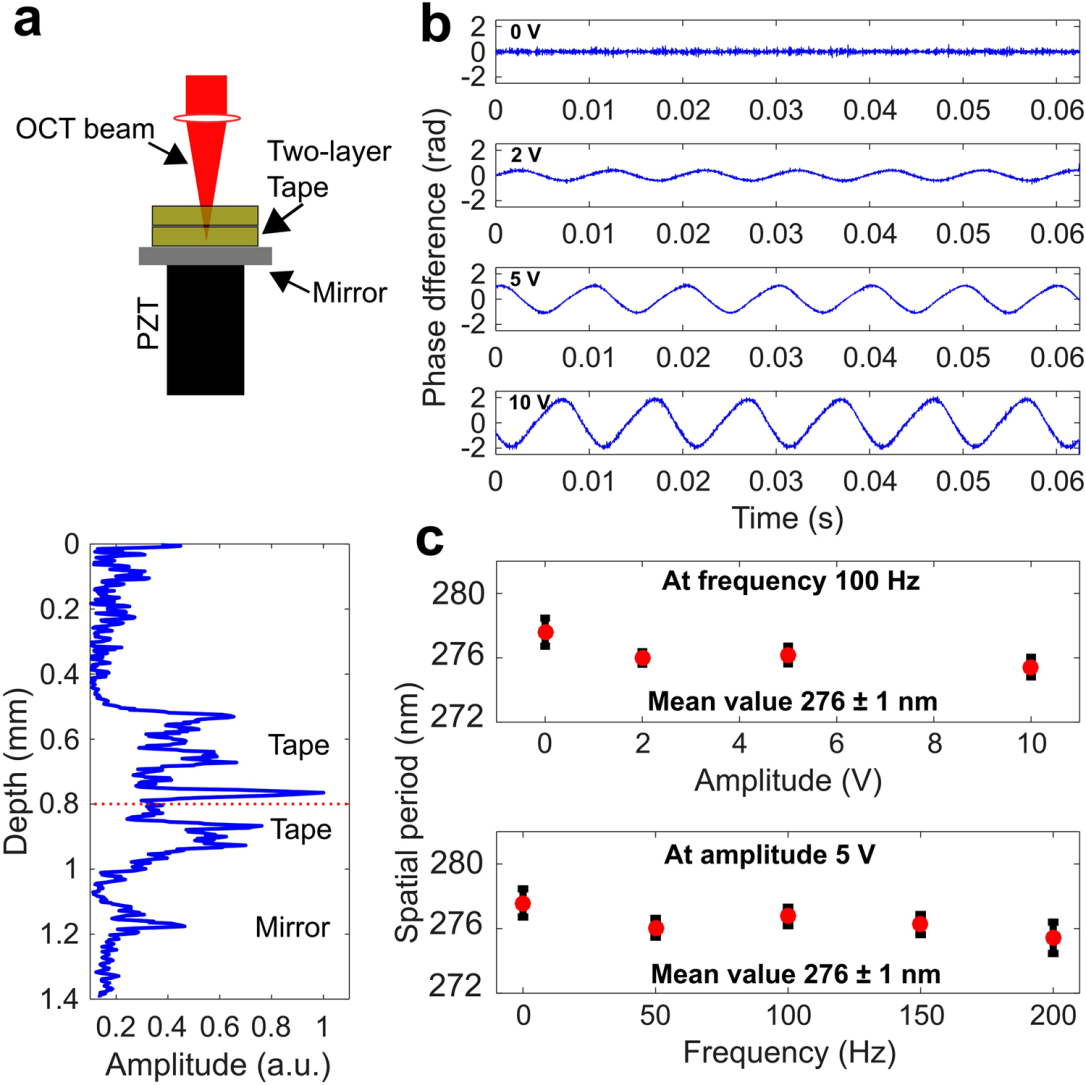
Phantom displacement study to characterize the effect of axial motion on nsOCT results. (**a**) Schematic and plot shows a two-layer tape phantom placed on top of a mirror and mounted on a PZT stack. (**b**) Phase difference plots of recorded scans at amplitudes of 0 V, 2 V, 5 V, and 10 V (from top to bottom). (**c**) Averaged spatial periods of the phantom as determined by the nsOCT algorithm for varying amplitudes and varying frequencies, respectively.

A multilayered tape phantom placed on a stack of lead zirconate titanate (PZT) (PE4, AE0505D16F, Thorlabs) was imaged with known displacements to determine if nsOCT processing (refer to Materials and Methods section) was sensitive to axial motion of the sample. The PZT material changes in thickness when an electric field is applied, which enables precisely controlled mechanical displacements. Figure 1(b) shows the measured phase difference at varying input voltage amplitudes (2 V, 5 V, and 10 V, which corresponded to axial displacements of 3 µm, 9 µm, and 17 µm, respectively) at a sine wave frequency of 100 Hz. At 0 V, no displacement was observed, which corresponds to the baseline phase stability of the system. The phase stability was determined by measuring the standard deviation of the phase difference signal, and was measured to be 19 ± 0.42 mrad. For increasing input voltage amplitudes, the phase difference method could clearly detect the increased axial motion with increasing voltage, as indicated by the measured sinusoidal pattern of the phase difference.

Next, nsOCT processing was applied to the same datasets. Figure 1(c, top) shows the measured spatial period for varying input voltage amplitudes at a frequency of 100 Hz. The results show less than a one-nanometer variation in the spatial period for all input voltage amplitudes. The measured mean value of all spatial periods was 276 ± 1 nm. Figure 1(c, bottom) shows the spatial period at varying actuation frequencies with a constant input amplitude of 5 V. A similar trend in the spatial period was observed, with minor deviation. This was further verified by measuring the mean values of the spatial periods and comparing them with the results from varying the amplitude.

**Figure 2:**
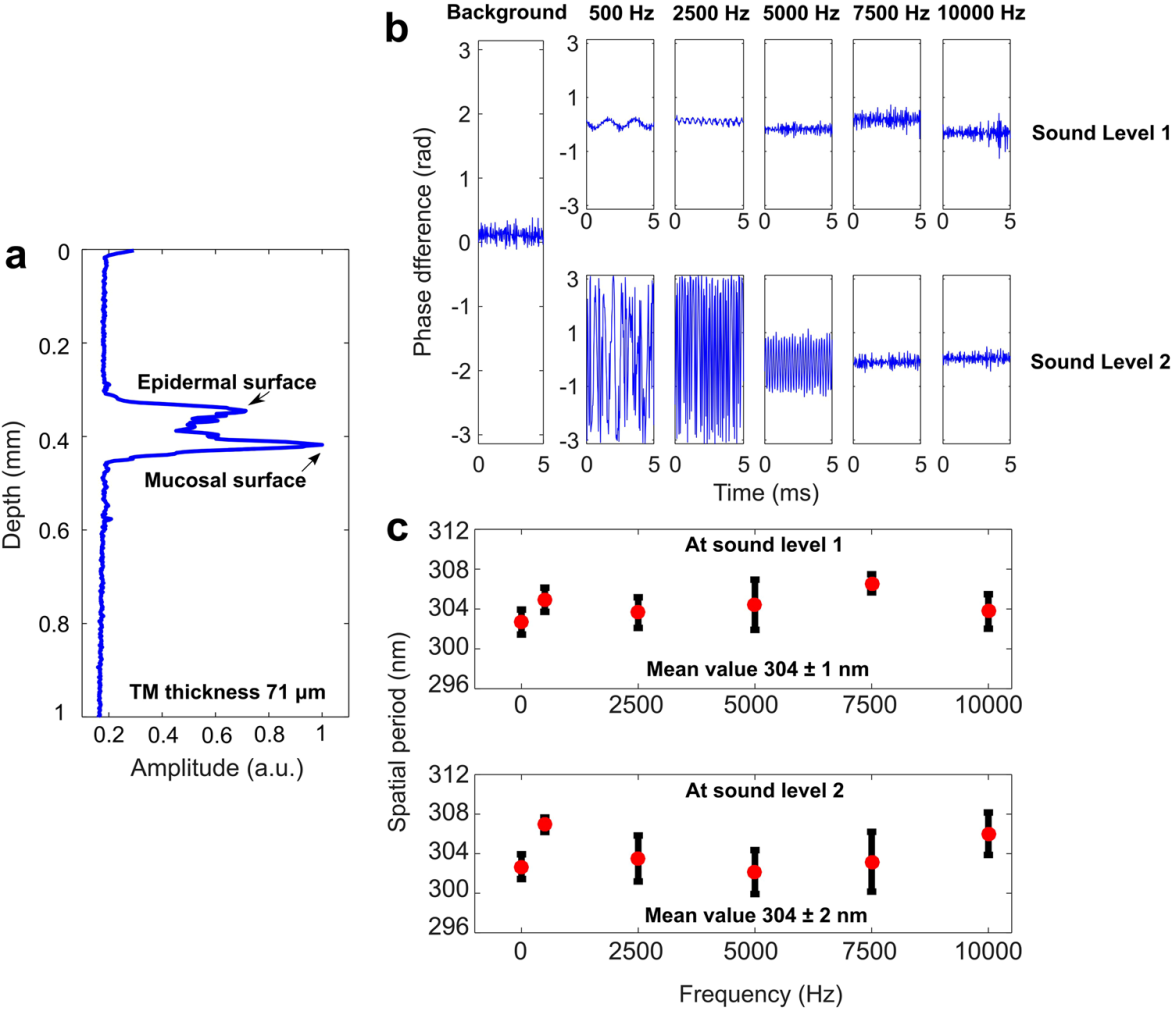
Axial motion detection of an *in vivo* normal human TM. (**a**) A-scan (depth-scan) of a healthy TM. (**b**) Phase difference plots of recorded A-scans at varying sound intensities and varying frequencies. (**c**) Averaged spatial periods of the TM at sound intensities and frequency.

Following the multilayered phantom displacement study, we imaged the *in vivo* TM displacements of a healthy subject. Figure 2(a) shows an axial A-scan collected *in vivo* from the TM of a healthy human volunteer. The peaks represent the two surfaces of the TM, giving a measured thickness of approximately 71 µm. Figure 2(b) shows the phase difference plots at two different sound intensities and at different frequencies ranging across 500 Hz, 2.5 kHz, 5 kHz, 7.5 kHz, and 10 kHz. The recorded sound intensity levels (in dB) are noted in Table 1. The phase difference plots clearly show that the TM was displaced for different sound intensities and frequencies, and generally followed the trend that pure tones at lower frequencies and higher intensities will vibrate the TM at greater amplitudes and induce these phase differences. Figure 2(c) shows the measured structural changes of the TM after nsOCT processing. Measured spatial periods showed a minor difference (mean spatial period 304 ± 1 nm) between the two different sound intensities and across the range of frequencies investigated. While each measurement was acquired sequentially in time during the experimental session, there may have been a slight offset in the probe beam position on the TM, which may be responsible for the marginal fluctuations in the measured spatial period. Nevertheless, the calculated mean spatial period for the two different sound intensities across the range of frequencies showed similar spatial periods (difference 0 nm, uncertainty 1 nm) and suggested that the nsOCT processing and results are not sensitive to axial displacement of an *in vivo* TM. Both experiments confirm our hypothesis that nsOCT is only sensitive to axial structural changes and not to the axial displacement of the sample, even under *in vivo* measurement conditions. This suggests that nsOCT can be used more practically in clinical settings without concern of confounding measurements.

**Table 1:** Recorded dB levels for corresponding frequencies at two different sound intensity levels.

Frequency (Hz)	Sound intensity (dB)		Intensity above background (dB)	
	Sound level 1	Sound level 2	Sound level 1	Sound level 2
Background noise level	44	44		
500	81	96	37	52
2500	93	110	49	66
5000	83	100	39	56
7500	76	95	32	56
10000	73	91	29	47

### Experimental validation and analysis of normal and abnormal TMs

**Figure 3:**
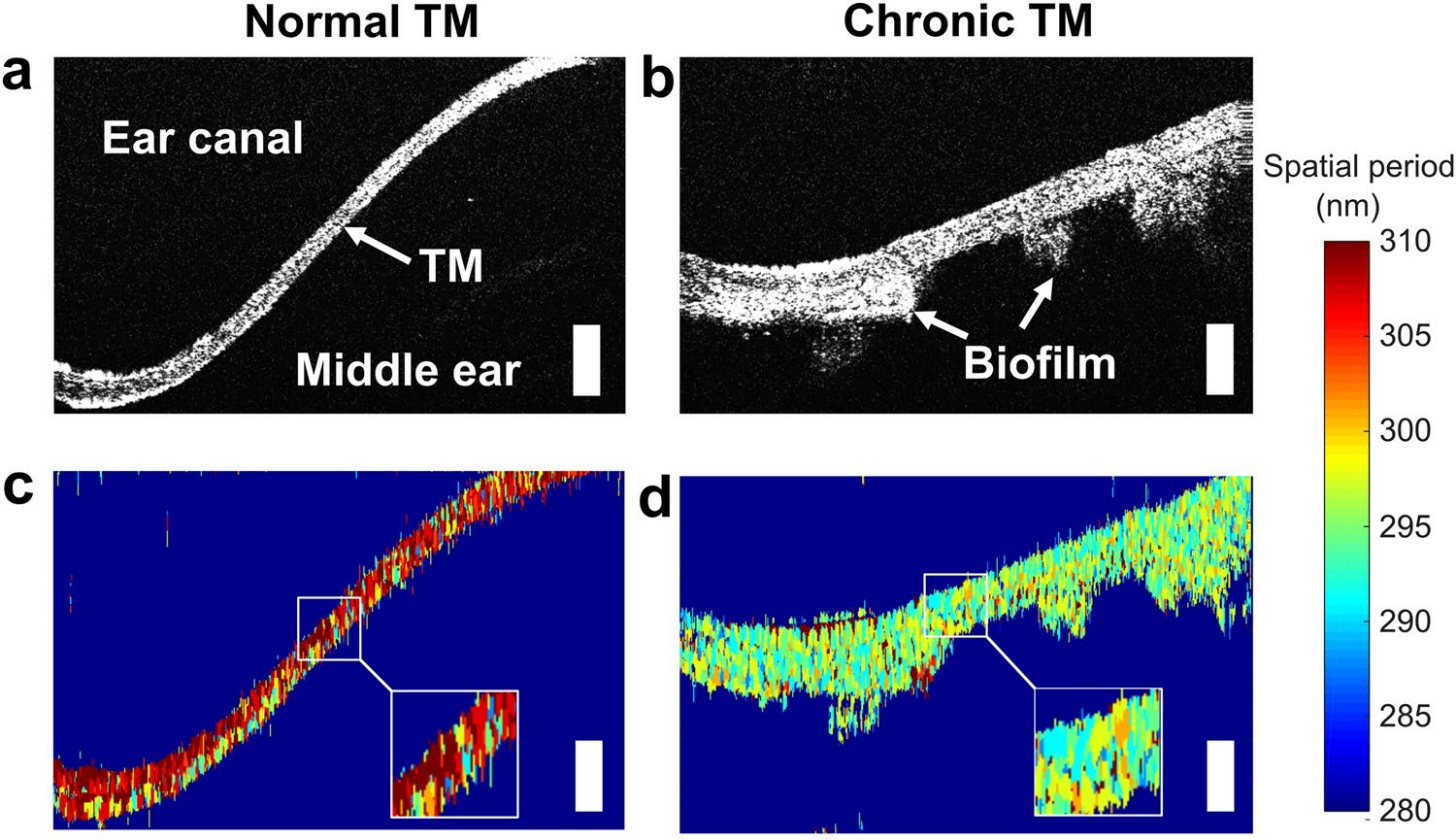
Representative OCT and nsOCT images of *in vivo* human TMs. Cross-sectional OCT images of TMs under (**a**) normal and (**b**) chronic OM conditions. Figures (**c** and **d**) show the corresponding nsOCT images. The color bar indicates the structural changes between 280–310 nm. The normal TM shows higher spatial periods compared to the abnormal TM, which can be seen more clearly in the inset figures. The white scale bars represent 300 µm in depth.

Representative OCT and nsOCT images of a normal TM and one associated with chronic OM are shown in Fig. 3. Figure 3(a) shows the cross-sectional OCT image of a normal TM with a thickness of approximately 105 µm. A cross-sectional OCT image of a TM in chronic OM with a thick accompanying biofilm is shown in Fig. 3(b), which reveals an increased thickness (ranging from 132 to 204 µm) when compared to the normal case.

The nsOCT image of the normal TM shows higher spatial periods, which indicates that the axial structures were more periodic in nature. For the TM in chronic OM, nsOCT showed a decrease in the spatial period from the periodic structures, and hence suggests that deformation or disruption of the structures have occurred at the nano-scale^2,14^. Further, an earlier report suggested that the histology of a normal TM has periodic structures, whereas, TMs in acute and chronic OM demonstrated significant structural changes that disrupt the periodicity of the laminar structure^2^. Our quantitative measurements of axial structural changes demonstrated with nsOCT are in good agreement with this report. The time required to process an nsOCT image was approximately 15 s per B-scan (2048 depth pixels × 1000 lateral pixels) on a high-end desktop PC.

Tables 2 and 3 summarize the data of 59 subjects (102 TMs) included in this study, such as relevant clinical history, measured TM thickness, and measured nsOCT value. Separation into the various subject groups (normal, acute OM, chronic OM) was determined by the measured TM thickness in OCT images. Previously, a quantitative study on *in vivo* human TM thickness showed these metrics differentiated the normal (105 ± 25 µm), acutely infected (173 ± 35 µm), and chronically infected (288 ± 76 µm) TMs in pediatric subjects^16^. We have adopted these metrics in our nsOCT algorithm to further classify the TMs between normal, acute OM, and chronic OM conditions.

**Table 2:** Summary of normal subjects, physician diagnoses, OCT thickness values, and corresponding nsOCT values.

Subject	Patient History	TM thickness (µm)	nsOCT value (nm)
Normal TM			
N1	Normal	106	310
N2	Normal	110	311
N3	Normal	143	305
N4	Normal	119	303
N5	Normal	106	306
N6	Normal	129	302
N7	Normal	127	308
N8	Normal	133	301
N9	Normal	121	305
N10	Normal	115	303
N11	Normal	133	303
N12	Normal	106	303
N13	Normal	67	305
N14	Normal	88	303
N15	Normal	106	307
N16	Normal	94	302
N17	Normal	131	300
N18	Normal	129	302
N19	Normal	113	306
N20	Normal	121	302
N21	Normal	102	301
N22	Normal	123	306
N23	Normal	92	301
N24	Normal	121	302
N25	Normal	88	306
N26	Normal	102	301
N27	Normal	125	307
N28	Normal	131	304
N29	Normal	102	302
N30	Normal	117	301
N31	Normal	109	303
N32	Normal	82	303
N33	Normal	104	302
N34	Normal	133	300
N35	Normal	116	305
N36	Normal	133	303
N37	Normal	94	303
N38	Normal	117	302
N39	Normal	108	303
N40	Normal	108	305
N41	Normal	84	303
N42	Normal	120	308
N43	Normal	101	303
N44	Normal	114	307
N45	Normal	98	301
N46	Normal	107	305
N47	Normal	127	305
N48	Normal	92	307
N49	Normal	111	301
N50	Normal	103	303
N51	Normal	111	303
N52	Normal	106	303

**Table 3:** Summary of imaging subjects with acute and chronic OM along with clinical history, OCT thickness values, corresponding nsOCT values, and additional notes.

Subject	Patient History	TM thickness (µm)	nsOCT value (nm)	Notes
Acute TM				
A1	Normal	161	299	
A2	Normal	173	297	
A3	URI	143	299	
A4	History of OM	172	299	
A5	Normal	165	297	
A6	Little sclerosis	171	300	
A7	Normal	194	298	
A8	URI	202	299	
A9	Normal	160	296	
A10	Normal	176	296	
Chronic TM				
C1	History of OM and tubes	193	299	Biofilm only
C2	Thickening of ear drums bilaterally	211	297	Effusion
C3	Effusion	207	298	
C4	History of OM and tubes	199	294	Biofilm only
C5	OME	292	297	
C6	OME	317	298	
C7	OME	281	297	
C8	Normal	196	299	Biofilm only
C9	OME	208	298	
C10	OME	208	300	
C11	OME	206	299	Effusion and biofilm
C12	OME	202	298	
C13	URI	203	298	
C14	OME	211	298	
C15	URI	223	297	
C16	Normal	238	298	Effusion
C17	Normal	204	299	Effusion and biofilm
C18	URI	240	300	
C19	History of OM	245	300	
C20	Normal	227	299	Biofilm only
C21	Normal	172	297	Effusion and biofilm
C22	OME	216	298	
C23	Normal	207	299	Effusion
C24	OME	266	299	
C25	OME	211	296	
C26	URI	282	298	
C27	URI with history of OM	224	297	
C28	OME	205	297	
Abnormal nsOCT				
AB1	Normal	129	297	
AB2	Normal	110	300	
AB3	Normal	137	299	Viral infection
AB4	Normal	142	296	
AB5	Normal	140	299	
AB6	Normal	129	299	
AB7	Normal	131	299	Right ear normal, but effusion on left ear
AB8	Normal	131	296	Viral infection
AB9	Normal	107	295	Viral infection
AB10	Normal	140	300	
AB11	Normal	115	298	
AB12	Normal	131	299	Viral Infection

OM = otitis media; TM = tympanic membrane; URI = upper respiratory infection; OME = otitis media with effusion; MEE = middle ear effusion; ASOM = acute suppurative otitis media.

**Figure 4:**
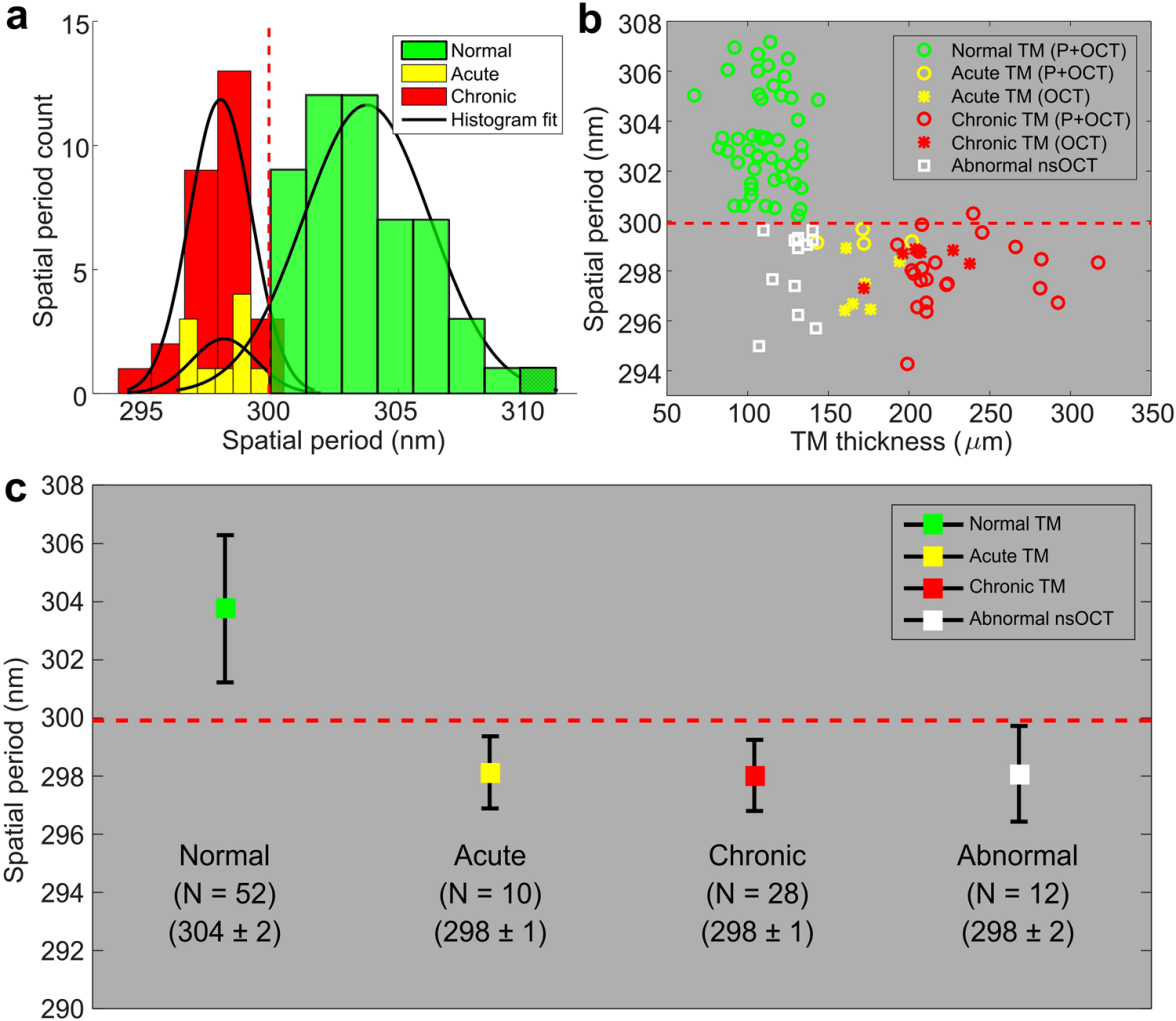
Quantitative analysis of TM nsOCT values based on measured TM thickness between groups. (**a**) Histogram plot and fit of TM nsOCT values. The intersection point (dashed vertical line) between normal and chronic OM TMs was selected as a threshold. (**b**) Representative OCT thickness and nsOCT values of TMs under normal (green), acute OM (yellow), and chronic OM (red) conditions, with means and standard deviations for each group plotted. Abnormal TMs were classified based on nsOCT values and did not agree with OCT-measured TM thicknesses and physician reports. (**c**) Measured average nsOCT values and statistical analysis presented by group.

A quantitative analysis of the TMs with the nsOCT method is summarized in Fig. 4. For this analysis, a square region of interest (ROI) approximately 200 × 200 pixels from each nsOCT image was manually selected, followed by averaging of the spatial period values within each ROI. Figure 4(a) shows a histogram plot and fit of nsOCT values for each of the three groups of TM conditions. The intersection point (dashed vertical line) between the normal and chronic OM histogram fitted traces was selected as a threshold to separate the groups (threshold ~300 nm). Figure 4(b) shows a scatter plot of the representative OCT thicknesses and nsOCT values for all of the TMs in this study. The data points shown in this plot are displayed with different markers: circles, asterisks, and squares. The circle data points represent TMs where the physician reports were in agreement with the OCT classifying algorithm based on the TM thickness (normal, acute OM, or chronic OM). The asterisk data points represent TMs where the physician reports were not concordant with the measured OCT thicknesses, and where OCT scans showed an increased thickness of the TM. Interestingly, the nsOCT values for the normal, acute OM, and chronic OM groups agreed with OCT thickness values and suggest that OCT and nsOCT measurements were more sensitive at identifying nano/micro structural changes in the TM as a result of the infection. The few datasets (*N* = *12*) shown as square data points indicate that the TM has nsOCT values similar to the acute OM and chronic OM cases, and hence are labeled as abnormal nsOCT. Contradictory to these nsOCT values, the physician assessment and the OCT data in these datasets suggested that the TMs were normal. As validated with normal, acute OM, and chronic OM cases, nsOCT detects nano-scale periodicity of the sample structure, authenticating that these changes plausibly correspond to the early nano-scale structural changes occurring in the TM as a result of the infectious process, such as inflammation, vascular dilatation, and cellular infiltrates and exudates. It is therefore suspected that the early nano-structural changes that occur in the TM begin with the acute infection, and persist throughout the transition to chronic OM. Correspondingly, mean and standard deviation values for each group are shown in Fig. 4(c).

The results of the statistical analysis are shown in Table 4. The statistical analysis shows that nsOCT metrics can differentiate normal TMs from the TMs in other groups (acute OM and chronic OM), respectively, (p < 0.001). The comparison between acute OM and chronic OM groups, however, are not statistically significant. The reason for this is not entirely clear, and further studies are required to investigate the pathological mechanisms that alter the nano-structural anatomy overtime as OM progresses from an acute infection to a chronic infection.

**Table 4:** Statistical results between normal, acute, chronic, vs. abnormal groups.

Comparison Group	*t* Value	*P* value
Normal and acute	6.9	<0.001
Normal and chronic	11.42	<0.001
Normal and abnormal nsOCT	7.48	<0.001
Acute and chronic	0.29	0.77
Acute and abnormal	0.11	0.92
Chronic and abnormal	0.14	0.89

### Longitudinal analysis of an ear infection with surgical intervention

**Figure 5:**
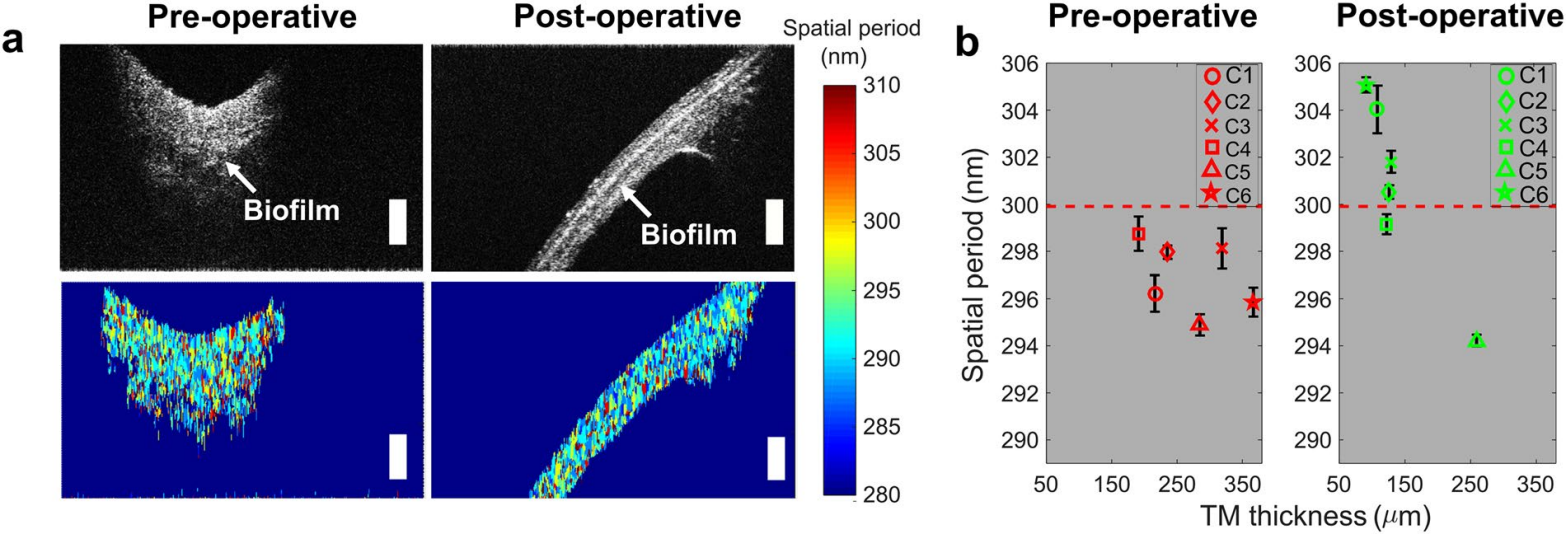
Longitudinal analysis of *in vivo* TM changes with surgical treatment of ear infection. (**a**) Representative cross-sectional OCT and nsOCT pre- and post-operative images from subject C5. (**b**) Means and standard deviations of OCT thicknesses and nsOCT values of all six interventional cases. Scale bars represent 300 µm in depth.

This study includes longitudinally analyzed datasets from pre- and post-surgical TMs in pediatric subjects undergoing surgery to treat chronic OM^23^. Table 5 summarizes the details of the subjects included in this study, along with their clinical history, and OCT and nsOCT measurements. These subjects were all diagnosed with chronic OM and were undergoing surgical placement of tympanostomy tubes in the TMs to surgically treat their infections. All six subjects were imaged pre-operatively, intraoperatively, and at their follow-up visits, which were approximately one month after their surgeries. In all of these subjects, OCT images revealed a biofilm affixed to the inner mucosal surface of the TM during their pre-operative and intraoperative imaging sessions. Subsequently, in 5 of the 6 subjects (83.3%), those subjects who had a successful outcome and resolution of their chronic OM after surgery, no biofilm was detected during their post-operative visit. Figure 5 shows the representative cross-sectional OCT, nsOCT, and corresponding metrics of this longitudinal analysis from the one subject (C5) who did not respond successfully to this surgical intervention. The pre-operative OCT image confirmed the persistence of the infection as indicated by the presence of a biofilm, and approximately one month after the surgery, the same subject was imaged at the otolaryngologist’s outpatient clinic. The OCT cross-sectional image of the TM clearly revealed the persistence of the biofilm. This may be due to either the persistence or recurrence of the infection, and likely is associated with an ineffective surgical treatment outcome. The nsOCT processing on these datasets showed a persistently low spatial period, which agrees with the OCT measurement.

**Table 5:** Summary of imaging subjects, clinical histories, TM thicknesses, and nsOCT values for pre-operative and post-operative time points.

Subject	Patient History	TM Thickness (µm)		nsOCT value (nm)	
		Pre-operative	Post-operative	Pre-operative	Post-operative
C1	Recurrent AOM and OME, repeated ABx therapy	216	108	296	304
C2	ETD and Chronic OME, Hearing Loss	235	126	294	303
C3	ETD and Chronic OME, Hearing Loss	319	129	292	302
C4	Recurrent AOM, ETD	191	122	293	299
C5	Recurrent AOM, ETD, Hearing Loss	285	259	295	295
C6	ETD and Recurrent AOM, repeated ABx therapy	325	91	290	305

ABx: Antibiotic, AOM: Acute otitis media, ETD: Eustachian tube dysfunction, OME: Otitis media with effusion.

Figure 5(b) shows the OCT and nsOCT metrics for all six subjects (six ears), both pre-operatively and post-operatively. Pre-operatively, all six subjects had increased TM thicknesses, which suggests the presence of biofilm. Imaging post-operatively, the thicknesses of the TMs were normal except for the one noted subject (C5). A similar trend was observed with the nsOCT metrics. For the nsOCT measurements, two cross-sectional nsOCT images were used. The maximum spatial period was obtained by averaging an ROI, and the uncertainty was measured by calculating the standard deviation of the ROI. Pre-operatively, nsOCT metrics showed a lower spatial period, which suggested the disruption and deformation of the TM structure, while the post-operative spatial periods were near or above the threshold level. Post-operatively, subject C5 showed a lower spatial period, which agreed with the OCT thickness measurement. For subject C4, OCT showed a marked recovery. However, the spatial period was close to but still below the threshold level, suggesting that this subject was responding to the surgical treatment, but that the abnormal nano-structural changes in the TM were likely still resolving.

## Discussion and Conclusion

Though the otoscope is used ubiquitously by physicians to diagnose middle ear infections, an accurate diagnosis relies largely on physician experience and expertise. The subjective qualitative visual information provided by the otoscope can lead to incorrect diagnoses that can affect treatment and outcomes. To address this issue, several clinical studies have used OCT to assist in the diagnosis of a middle ear infection, largely because OCT can image sub-surface features in the middle ear, primarily effusions and biofilms^15,17^. Previously reported studies showed that OCT could clearly differentiate between normal TMs and those associated with acute and chronic OM^16^. However, the structural information provided by OCT is limited to the micron-scale. It is evident from histological images^2,14^ that the TM undergoes significant structural changes at the nano-scale, which cannot be detected by standard OCT, but may have diagnostic and/or prognostic value. To address the need for increased sensitivity to early nano-scale axial structural changes in the TM, we have used nsOCT to measure the nanometer periodicity of the sample structure. In this study, we also used OCT-measured TM thickness metrics to differentiate between various affected groups. Together, we report the unique ability to detect the nano-structural changes of the TM that occur at the early stages of OM.

The analysis of our experimental human subject data can be associated with the structural changes of the TM. Broadly, OCT measurements are related to the optical scattering properties of the TM. The normal TM is expected to be thin, have a thickness of approximately 100 µm, and contain uniform, highly scattering micro-structure. In the case of an acute ear infection with inflammation, the TM will thicken, compared to the normal TM, and produce a lower optical scattering signal likely due to the dilution of the scatterers in the TM from the influx of extracellular fluid during the inflammatory process. In chronic OM, the scattering of the TM appears to be similar to that of the normal TM. Although overall thickness, including biofilm, will significantly increase, the thickness of the TM alone will return to that of a normal ear (Fig. 3b), which was also previously reported^16^. Interestingly, when compared to the nsOCT image of a chronic OM case, the axial structure of the TM alone showed a lower spatial period (Fig. 3d). This suggests that the nano-structure of the TM along with biofilm remains altered, and this information was not available from the OCT image data alone. Moreover, OCT measurements showed that 64 of the TMs were normal, while 10 and 28 of the TMs were identified with acute OM and chronic OM, respectively. When comparing these statistics with nsOCT measurements, both acute OM and chronic OM cases showed a lower spatial period and agreed with the OCT measurements. However, out of the 64 normal TMs, nsOCT measurements identified that 12 of the normal TMs had a lower spatial period. These measurements suggest that the structure of these TMs was not periodic, and reveal early nano-structural changes in these TMs.

Abnormal nsOCT images of the TM consisted of either acute OM or chronic OM cases, as nsOCT alone could not statistically differentiate these two groups. These cases were also correlated with the clinical history of these subjects, which were diagnosed as normal TMs (Table 3). However, the physician reports for 4 of these subjects confirmed that these subjects had been diagnosed with a viral infection. It is clear from the U.S. Centers for Disease Control and Prevention (CDC) that apart from bacteria, viruses such as rhinoviruses, influenza, and adenoviruses may also cause AOM^24^. We believe our measurements confirm the early detection of the ear infection, even for subtle nano-scale changes induced by the inflammatory condition of a viral ear infection. Further study, however, is needed and should include a larger subject population before suggesting the potential use of this method for clinical diagnoses and medical decision making.

A longitudinal analysis study was performed to evaluate nsOCT changes in the TM after surgical treatment of chronic OM. In this study, tympanometry tube placement in the TM was effective in 5 out of 6 subjects who underwent surgery and had no recurrence of symptoms thereafter. Both OCT and nsOCT measurements observed a significant difference between pre- and post-operative timepoints. For one subject (C5), however, both nsOCT and OCT confirmed the persistence of nano-scale changes in the TM, along with a persistent biofilm, which is consistent with the fact that this subject did not respond appropriately to the surgical intervention.

There are a few challenges and limitations associated with the current nsOCT processing that need to be addressed before its quantitative use in larger clinical investigations. Our presented results showed that nsOCT could not differentiate between acute and chronic OM groups. This remains unclear as the thickness measurements showed the larger structural differences between acute and chronic OM cases, and were statistically different. We believe that the nano-structural changes in the TM that begin in an acute infection persist while the disease progresses to a chronic state. Detecting these nanostructural changes between the acute and chronic stages of this infectious process will help elucidate the structural alterations occurring in OM. Given the obvious limitations associated with invasive sampling of TMs for electron microscopy, these changes could be investigated by inducing and longitudinally tracking ear infections and middle-ear biofilms in the standard chinchilla animal model^25^. Finally, there is an inherent trade-off between resolution and sensitivity for detecting these structural changes. In conventional OCT, a two-dimensional image is commonly obtained with a fixed focal depth and a relatively large depth-of-focus, as the image quality (transverse resolution) degrades away from the focal plane. Similarly, this may also limit the accurate representation of an nsOCT image due to the inaccurate reconstruction of spatial frequencies. Computational imaging techniques such as interferometric synthetic aperture microscopy (ISAM) or computational adaptive optics (CAO) eliminate the trade-off between transverse resolution and depth-of-field, and provide spatially-invariant transverse resolution^26,27^. The computational ISAM and CAO imaging techniques reconstruct the Fourier spectra of spatial frequencies more accurately than conventional OCT, and hence may provide more accurate formation and representation of the nsOCT images.

In summary, this study presents a novel, noninvasive, and quantitative method to measure nano-structural changes of the *in vivo* human TM. Our results demonstrate that nsOCT detects the early nano-scale structural changes that occur when a normal TM becomes infected in OM. Future investigations are needed to determine the precise pathological mechanisms responsible for the similar spatial periods observed for both acute and chronic OM cases. Previous OCT-based ear imaging studies have shown a strong potential for clinical use and impact. In addition to the already established use of OCT for structural imaging in the ear, incorporating the additive information from nsOCT would enhance the detection sensitivity for the changes in the TM associated with this highly prevalent disease. This combinational method will not only generate quantitative information about both the TM thickness and the underlying nano-structural changes that occur during this infectious process, but will also provide additional information to the physician that can be used to improve diagnostic accuracy and clinical decision making.

## Materials and Methods

### Portable spectral-domain OCT system

**Figure 6:**
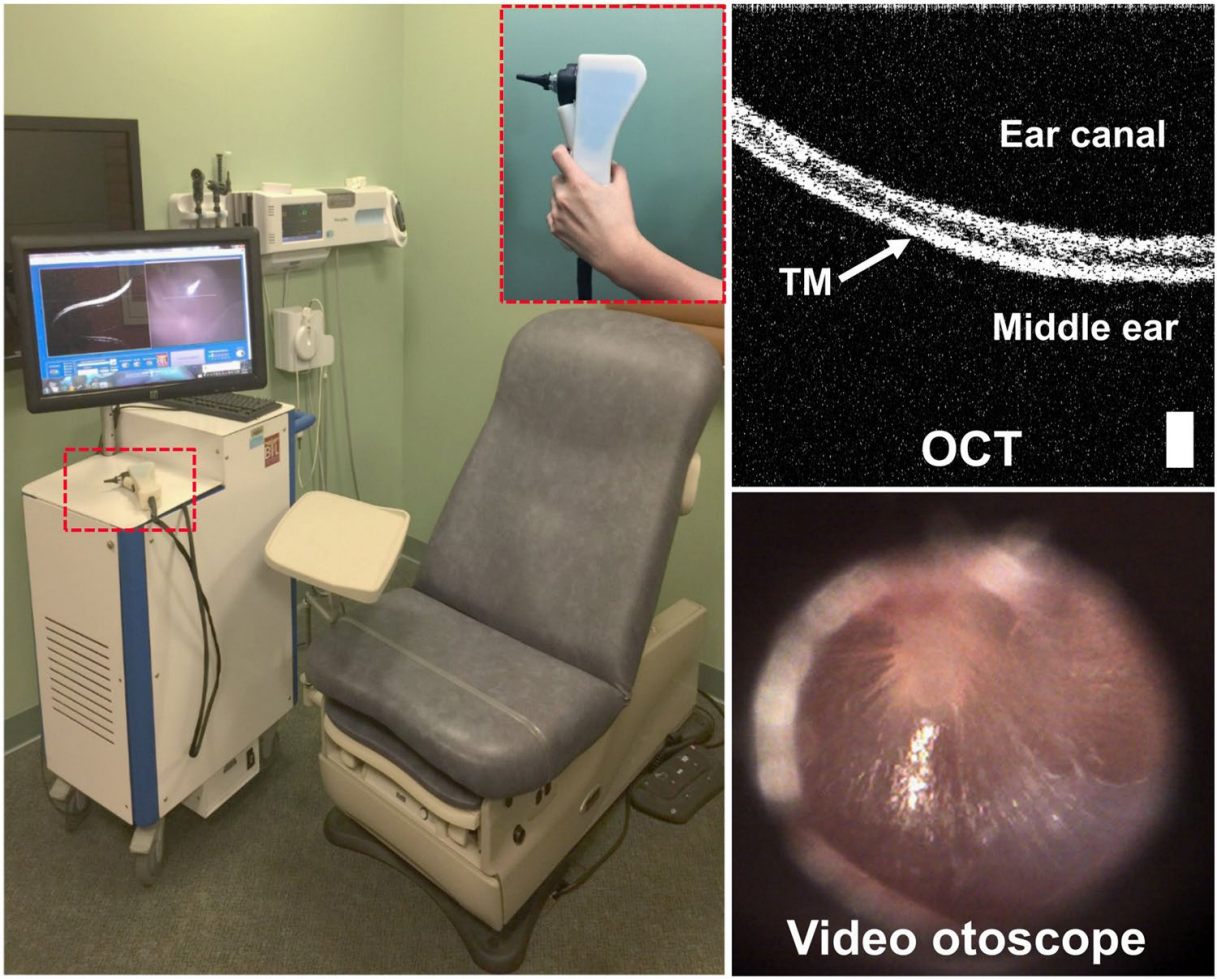
Photograph of the portable cart based SD-OCT system and handheld scanner (inset). The system was custom developed and can simultaneously acquire both a cross-sectional OCT image and a surface video image of the TM. Scale bar represents 100 µm.

A custom-built portable OCT system with a handheld probe (Fig. 6) was used to perform phantom experiments and image human subjects. More details on the system design can be found in a previous publication^13^. In brief, the system utilized a broadband superluminescent diode (SLD; T-860HP, Superlum, Ireland), centered at 860 nm with a bandwidth of 135 nm at full width half maximum. Cross-sectional images with 2048 depth pixels and 1000 lateral pixels (A-lines) were acquired using a line scan camera based spectrometer (C840-USR-00069, Wasatch Photonics, USA) at approximately 30 frames per second.

The handheld probe housed the sample arm optics along with a MEMS scanner (Advanced MEMS, USA) for lateral beam scanning and additional optics for video otoscope imaging of the TM surface. The axial and lateral OCT imaging resolutions of the system were measured to be ~2.4 µm and ~15 µm, respectively, in air. The data was acquired and displayed through a custom-designed graphical user interface (LabVIEW) and saved on a hard drive for nsOCT processing.

### Nano-sensitive optical coherence tomography

Nano-sensitive OCT is a computational variant of OCT which detects the spatial and temporal structural changes within three-dimensional scattering objects. The nsOCT method used in this manuscript was adapted from a spectral encoding of spatial frequency (SESF) approach which enables visualization of the structural changes of the objects with nano-scale sensitivity^28–30^.

**Figure 7:**
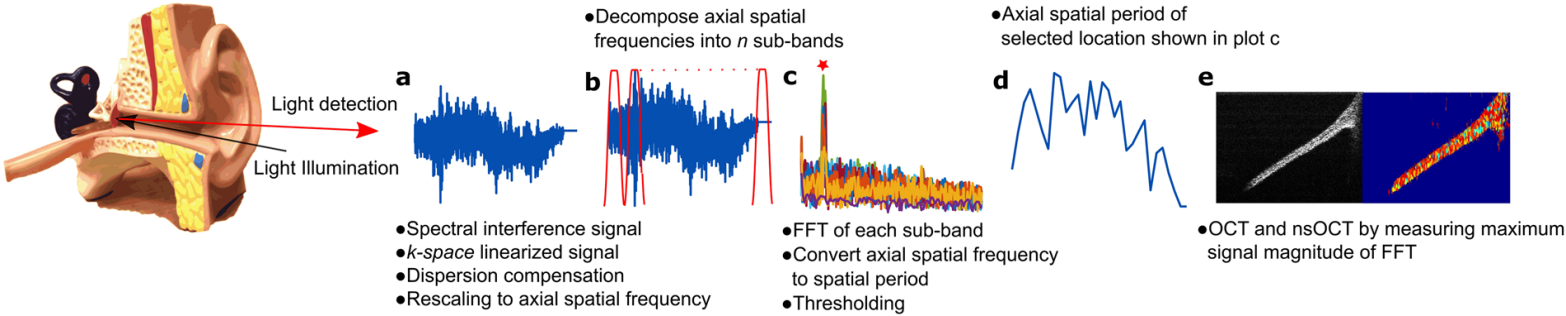
Flowchart for nsOCT signal processing. (**a**–**e**) Steps describe how the spectral interference signal results in the nano-scale structural changes of the sample.

Figure 7 outlines a flow chart for reconstructing an nsOCT image of a TM. A recorded spectral interference signal is rescaled from wavelength to frequency (*k-space* linearization) and then corrected for dispersion. To compute the nsOCT signal, the corrected complex amplitudes of the signal are converted to complex amplitudes of spatial frequencies (${V}_{z}$) based on the following relation:

1 $${V}_{z}=\frac{2}{\lambda }$$

where *λ* is the center wavelength of the light source. The range of the spatial frequencies is subsequently calculated as:

2 $${\rm{\Delta }}{V}_{z}=\frac{2{\rm{\Delta }}\lambda }{{\lambda }_{1}{\lambda }_{n}}$$

where Δ*λ* = *λ*_*n*_ − *λ*_1_ is the bandwidth of the light source and *λ*_1_ and *λ*_*n*_ are the shortest and longest wavelengths of the source spectrum.

The rescaled spectrum of complex amplitudes of spatial frequencies is then decomposed into multiple sub-bands. There is an inherent trade-off between the width of the sub-bands and the axial size of the voxel. In our processing, we have implemented 15 sub-bands for a total imaging depth of approximately 3 mm. Next, the A-scans of each sub-band are reconstructed using a Fast Fourier Transform (FFT), and then the maximum energy contribution (spatial frequency of maximum amplitude) for each sub-band for a given point is measured. The relation between the spatial periods (${{\rm{\Delta }}H}_{z}$) of the structure and the spatial frequencies are as follows:

3 $${\rm{\Delta }}{H}_{z}=\frac{1}{{\rm{\Delta }}{V}_{z}}.$$

An amplitude signal threshold of 40% was empirically selected to eliminate background noise. The scaled axial spatial periods were then corrected for the index of refraction, *n* = 1.52 for the tape phantom (measured experimentally as described by Meng *et al*.^31^) and *n* = 1.44 for the TM^32^. The measured spatial period resulting from the maximum signal amplitude directly corresponds to the dominant size of the structure.

### Displacement stability study

The objective of this initial study was to determine if the axial motion of the TM would affect nsOCT processing. We hypothesized that nsOCT would only be sensitive to axial structural changes and not to axial displacement under *in vivo* conditions. To validate the axial motion displacement, a phantom consisting of two layers of semi-transparent adhesive tape (3 M Scotch Magic Tape) was placed on a reflective mirror substrate (Fig. 1(a)). The phantom was subsequently mounted on a PZT stack, which when driven by a voltage waveform, generated a known axial displacement.

A total of 2000 M-scans (repeated A-lines from the same lateral position over time) were acquired from the phantom by fixing the handheld probe (Fig. 6) on a mount. Both the probe and phantom were placed on a vibration-isolated optical table. The axial motion of the PZT was first verified by an optical phase difference method^33^. The phase information of the spectral interference signal was then extracted from the FFT of each A-line and the phase difference between sequential A-lines was determined.

Following the phantom experiment, the nsOCT system was used to image the TM of a healthy subject in a quiet room. The TM was subsequently acoustically stimulated by a loud speaker (Dell, Model: A215) that was placed 5 cm from the ear, in the same horizontal plane. The loud speaker was connected to the auxiliary output of the computer and custom code written in LabVIEW was used to generate tones of varying frequency and amplitude. The data were recorded at two different sound intensities and across a range of frequencies including: 500 Hz, 2.5 kHz, 5 kHz, 7.5 kHz, and 10 kHz. A total of 160 M-scans (~5 ms) were acquired from the same lateral position on the TM and the corresponding phase differences and nsOCT values were measured.

### Data collection

Clinical OCT images of *in vivo* human TMs were retrieved from a database of previously collected and organized OCT images, digital otoscopy images, de-identified clinical reports, and de-identified human subject history information. All clinical image data were collected with the OCT system previously described and shown in Fig. 6. The data was collected over a two-year time period under an Institutional Review Board (IRB) protocol approved by Carle Foundation Hospital (Urbana, IL) and the University of Illinois at Urbana-Champaign (Urbana, IL), with subjects providing both informed consent and assent. All experiments were performed according to Declaration of Helsinki guidelines and regulations. Data was collected at multiple clinical sites, including the primary care physician’s office, the otolaryngologist specialist’s clinic, and an outpatient surgical center. The dataset for this study included 102 ears (from 59 pediatric subjects) grouped as normal ears (64), ears with acute OM (10), and ears with chronic OM (28). For a separate longitudinal study that included surgical intervention to place tympanostomy tubes in the TM to treat chronic OM, a dataset from 6 pediatric subjects with pre- and post-surgery image data were used for this processing. All subjects were less than 18 years of age, and no exclusions were made based on gender or race.

Image collection protocols were site specific and dependent upon the existing clinical workflow. At the primary care clinic and otolaryngologist specialty clinic, imaging was performed after routine ear examination. This allowed the physician to formulate their clinical impressions free of bias, and to avoid interruption of the standard-of-care procedures. For data collected at the outpatient surgical center, subjects were consented in the preoperative staging area and imaged intraoperatively after the subject was placed under anesthesia. The standard-of-care surgical procedure immediately followed imaging. At all sites, OCT imaging required no longer than 5 minutes for both ears. In the cases where long-term longitudinal follow-up was possible, imaging took place as the subject returned for and received care as per standard practice, typically starting at the specialist’s office, then at the surgical center, and finally for post-surgical follow-up back at the specialist’s office. More in-depth explanations of specific data recording protocols are described in prior publications^16,18^.

### Statistical analysis

The thickness of the TM was calculated from the average of three different locations for each cross-sectional OCT image and then corrected for the index of refraction of *n* = 1.44^32^. For the statistical significance of these measurements, a two-tailed Welch’s *t* test was performed between the normal, acute OM, and chronic OM groups. A significance level of 0.05 was used for all statistical tests. The statistical calculation was implemented in Matlab using the *ttest2* function.

### Data availability

The data that support the findings of this study are available from the corresponding author upon reasonable request, and for collaborative research projects.
